# Primary melanoma of the adrenal gland: a case report and review of the literature

**DOI:** 10.1186/1752-1947-5-273

**Published:** 2011-07-02

**Authors:** Luis González-Sáez, Salvador Pita-Fernández, Maria José Lorenzo-Patiño, Francisco Arnal-Monreal, José Machuca-Santacruz, José Romero-González

**Affiliations:** 1 Department of Surgery, University Hospital Complex of A Coruña, C/As Xubias de Arriba, 84, E-15006 La Coruña, Spain; 2 Department of Epidemiology, University Hospital Complex of A Coruña, C/As Xubias de Arriba, 84, E-15006 La Coruña, Spain; 3 Department of Pathology, University Hospital Complex of A Coruña, C/As Xubias de Arriba, 84, E-15006 La Coruña, Spain

## Abstract

**Background:**

Primary melanoma of the adrenal gland is exceptionally rare as demonstrated by the few cases reported in the medical literature, and it has a high fatality rate. We present the case of a patient with two relapses and survival to date.

**Case report:**

We describe the case of a 58-year-old Caucasian woman who consulted her doctor with symptoms of asthenia, anorexia and weight loss. A mass was palpated in her abdomen at the height of the left hypochondrium. A computed tomographic scan revealed a retroperitoneal mass measuring 10 cm × 15 cm originating in the left adrenal gland. A left nephroadrenalectomy and splenectomy were performed. Histopathologically, the retroperitoneal mass corresponded to a melanoma, and no primary melanoma was found in any other location. The patient was treated with interferon-α-2b. Three years after her diagnosis the patient presented with a retroperitoneal relapse of the mass measuring 7.2 cm, which was removed. Five years after the first relapse a new retroperitoneal relapse mass was diagnosed, which was also removed. Since then the patient has been healthy and free from illness.

**Conclusion:**

Histological and immunohistochemical studies, together with the criteria described by Ainsworth *et al. *and Carstens *et al*., allowed us to diagnose primary melanoma of the adrenal gland.

## Background

Primary melanoma of the adrenal gland is an exceptionally rare occurrence, as demonstrated by the few cases described in the medical literature [1-11]. Both primary and metastatic melanomas of the adrenal gland are rarely clinically evident, and in most cases they are incidental findings. Generally, the signs and symptoms that characterize these clinical entities are not at all specific, with pain being the most common manifestation, together with imprecise gastrointestinal disorders, caused by the compression of the structures adjacent to these neoplasias [12]. It is not unusual to come across the typical symptoms of any tumor process, such as asthenia, anorexia and weight loss.

Primary melanoma of the adrenal gland is usually a voluminous, non-functional tumor showing heterogeneous contrast enhancement on the computed tomographic (CT) scan. The diagnosis is made on the basis of histological and immunohistochemical studies.

The adrenal glands can be sites for the metastatic dispersal of cutaneous or visceral melanomas in up to 50% of cases [13], and histological and immunohistochemical studies do not make it possible to differentiate metastatic tumors from primary melanomas. In 1976, Ainsworth *et al. *[14] established a series of criteria for establishing these differences. These criteria are (1) a detailed clinical record indicating no prior existence of cutaneous melanomas or cutaneous lesions that may have reappeared, (2) careful cutaneous and ocular exploration to eliminate the presence of lesions, (3) an exhaustive evaluation to eliminate any other visceral location, (4) a pattern of recurrence concordant with the location site and (5) from the histological point of view, non-typical melanocytes are usually seen on the periphery of the lesions, which does not occur with secondary melanomas. Subsequently, in 1984, Carstens *et al. *[4] established other, similar criteria which make it possible to diagnose a primary melanoma of the adrenal gland once the pertinent clinical, histological and immunohistochemical studies have been carried out. These criteria are (1) neoplastic involvement of a single gland; (2) absence of melanoma in the rest of the organism; (3) absence of previous excisions of pigmented mucous, cutaneous or ocular lesions; and (4) exclusion of any hidden pigmented lesion, preferably by autopsy. Our present case meets the Carstens *et al. *criteria, with the exception of the last one, since our patient is still alive.

Primary involvement of the adrenal gland is extremely rare. Only 23 patients have been described in the English-language literature, and documentation was complete in only 11 of them [1-11] (Table 1).

**Table 1:** Reported cases of primary malignant melanoma of the adrenal gland

Reference	Year	Age	Sex	Location	Size	Clinical data	Treatment	Follow-up
Kniseley and Baggentoss [1]	1946	60	M	Left	8.5 cm × 4.5 cm × 4.5 cm	Back pain, abdominal mass, anorexia	Radiotherapy	Died two months after detection
Dick *et al. *[2]	1955	62	M	Right	17 cm × 12 cm × 10 cm	Painless, slowly growing mass	Surgery without specification	Died 15 months later
Sasidharan *et al. *[3]	1977	52	F	Left	13 cm × 10 cm × 5 cm	Asymptomatic; Well-defined, partly mobile, non-tender, globular lump confined to the left lumbar region	Adrenalectomy and nephrectomy	No follow-up
Carstens *et al. *[4]	1984	32	F	Left	14 cm × 9 cm × 5 cm	Pain in the left flank, weight loss	Adrenalectomy and nephrectomy	Died seven months later
Parker and Vincent [5]	1986	77	M	Right		Asymptomatic. Casual finding in prostatic patient. Extrinsic compression of the proximal right ureter and displacement of the collecting system and renal contour laterally.	Autopsy examination	Died two months after diagnosis
Dao *et al. *[6]	1990	46	F	Right	8 cm × 7 cm × 6 cm	Severe right flank pain	Adrenalectomy and nephrectomy	Died one month later
Amérigo *et al. *[7]	2000	24	F	Left	9 cm	Intense post-prandial epigastric pain and weight loss	Adrenalectomy and nephrectomy	Alive 46 months later
Zalatnai *et al. *[8]	2003	41	F	Left	7.3 cm × 6 cm	Nausea, mild weight loss, abdominal tenderness and constant left flank pain	Explorative laparotomy (non-resectable tumor)	Died three months later
Granero *et al. *[9]	2004	78	M	Right	11 cm	Right flank pain, fever, nausea and vomiting	Adrenalectomy, nephrectomy and splenectomy	Alive 12 months later
Liatsikos *et al. *[10]	2006	42	M	Left	5.5 cm × 6 cm	Mild flank pain, weight loss and anemia	Laparoscopic trans-peritoneal adrenalectomy	Alive 12 months later
Bastide *et al. *[11]	2006	33	M	Right	-	Right paroxystic lumbar pain, mass in the right flank	Adrenalectomy and nephrectomy	Died 18 months after surgery
Present case	2009	58	F	Left	16 cm × 11 cm	Asthenia, anorexia, weight loss, left abdominal mass and anemia	Adrenalectomy, nephrectomy and splenectomy	Local recurrence, alive 10 years after first surgery
Summary statistics		50.4 ± 17.2 Median = 49	M = 50.0%	Left = 58.3%	10.9 cm ± 3.8 cm Median = 10 cm Range = 6 cm to 17 cm			

In 1946, Kniseley and Baggentoss [1] published a case and reviewed 11 cases known at that time, which had not been completely researched because they failed to meet the diagnostic criteria that were subsequently established. In 1955, Dick *et al. *[2] published a new case of melanoma of the adrenal gland that occurred together with a pheochromocytoma, establishing the histological differentiation between the two. Twelve years later Sasidharan *et al. *[3] published an article describing a primary melanoma of the adrenal gland in a 62-year-old woman that was discovered during a routine medical examination. In 1984, Carstens *et al. *[4] reported a case of a primary melanoma of the adrenal gland and another of the lung, on the basis of which they established a series of criteria for differentiating primary melanoma of the adrenal glands from metastatic melanomas. In 1990, Dao *et al. *[6] described two cases, although only one of them met the criteria for primary melanoma of the adrenal gland. They also reviewed the literature and included four well-documented cases: those published by Kniseley and Baggentoss [1], Dick *et al. *[2], Sasidharan *et al. *[3] and Carstens *et al. *[4]. In 2000, Amérigo *et al. *[7] reported a new case and reviewed the literature, including five well-documented cases that had already been described. Up until that time, there had been six perfectly documented cases. To these well-documented cases, we add the case reported by Parker and Vincent [5] in 1986, which had not been described in the previous reviews. We include this case because the CT scan, histopathology and meticulous post-mortem study indicated that it met the criteria of Carstens *et al. *[4]. In 2003, Zalatnai *et al. *[8] described a primary melanoma of the adrenal gland in a 44-year-old woman. In 2004, Granero *et al. *[9] described another case in a 78-year-old man. Two years later, in 2006, two more cases were published: one by Liatsikos *et al. *[10] in a 42-year-old man and another by Bastide *et al. *[11] in a 33-year-old woman. We therefore have gathered 11 well-documented case reports, which, together with our present case, makes a total of 12 (Table 1).

The mean age of the patients in all of these 12 cases is 50.4 ± 17.2 years, with a median age of 49 years and an age range between 24 and 78 years. Fifty percent of these patients were men, and the initial mean size of the tumors was 10.9 cm ± 3.8 cm with a median size of 10 cm and a range between 6 cm and 17 cm. The most frequent symptom in these cases was pain (Table 1). Apart from these cases described in the English-language literature, we found two cases in the Spanish-language literature, one of which was described by Deus Fombellida *et al. *[12] and involved a 46-year-old woman, and another described by Rodriguez Antolín *et al. *[15] which involved a doubtful case in a 50-year-old man, in which it is not clear whether the tumor was primary or metastatic.

In this article, we report a new case of primary melanoma of the adrenal gland diagnosed on the basis of histological and immunohistochemical studies and after having excluded the presence of hidden pigmented lesions. In 11 years of follow-up after the appearance of the lesion, melanoma has not been found in any other part of the patient's body, which allows us to eliminate a secondary or metastatic origin of the lesion.

Primary melanoma of the adrenal gland is treated by surgery involving the complete removal of the tumor and, nearly always, the removal of the adrenal gland and kidney. Primary melanoma of the adrenal gland has a high fatality rate; the patient described by Amérigo *et al. *in 2000 [7] was still alive after 46 months of follow-up, at the time the clinical case was published, and this patient is still alive today. The patients in the rest of the published cases survived less than 19 months after surgery.

## Case presentation

A 58-year-old Caucasian woman consulted her doctor with symptoms of asthenia, anorexia and weight loss 15 days after they first appeared. In her physical examination, pallid skin and mucous membranes were observed. Her abdomen was soft and yielding and tender to deep palpation of the left hypochondrium, where a mass could be felt.

**Figure 1:**
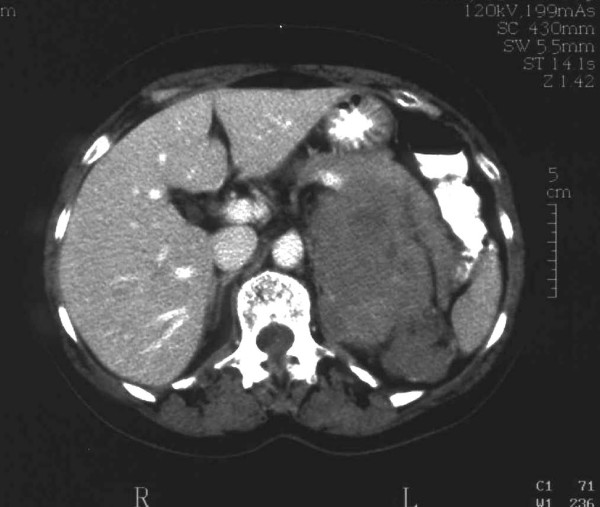
**Tumor measuring 10 cm × 15 cm in the upper region of the peri-nephric space**. The tumor's origin appears to be in the left suprarenal gland, which is surrounded by the mass.

In the medical tests carried out, the only altered parameters were hematocrit 24%, hemoglobin 8 g/dL, red blood cell count 2,500,000/mm^3 ^and blood glucose 152 mg/dL. An abdominal ultrasound revealed a mass with a diameter of approximately 10 cm at the area between the tail of the pancreas, the left adrenal gland and the upper pole of the left kidney. An abdominal CT scan confirmed the presence of a large retroperitoneal tumor measuring 10 cm × 15 cm that seemed to have its origin in the left adrenal gland, which was surrounded by the tumor, although the possibility of another type of primary retroperitoneal tumor could not be excluded (Figure 1). On the basis of these findings, an intravenous pyelogram with contrast dye was obtained, which revealed only a slight displacement of the left kidney in relation to the described tumor. A chest X-ray did not reveal any significant alterations.

We decided to perform surgery with the patient under general anesthesia. We made a bilateral subcostal incision and found a large retroperitoneal tumor in the left hypochrondrium with a grooved surface and a diameter of approximately 15 cm. The tumor was removed, including the kidney and left adrenal gland, together with the spleen and greater omentum. The patient recovered without any post-operative complications.

The anatomopathological study corresponded to a tumor weighing 625 g and measuring 16 cm × 11 cm with a nodular, fleshy appearance and areas of hemorrhage and necrosis. The microscopic study showed a tumor with fusiform and ephithelioid cells, with frequent multi-nucleation, monstrous nuclei, intra-nuclear inclusions, prominent nucleoli and a high number of mitoses, as well as a notable lymphocytic infiltrate. On the periphery of the tumor, remnants of the adrenal gland were recognizable, with tumor cells that had infiltrated the wall of the large venous vessels.

**Figure 2:**
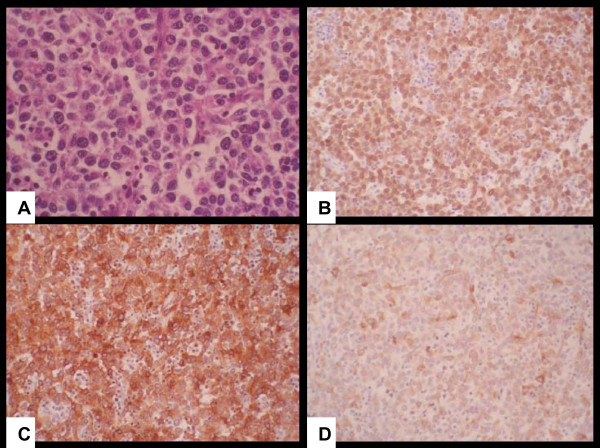
**(A) Histopathology of the primary and recurrent tumor showing a solid mass with nests of cells with an epithelioid appearance and eosinophilic cytoplasm (hematoxylin and eosin stain; original magnification, × 400)**. Immunohistochemical stains for S-100 protein showed **(B) **an intense diffuse positivity, and **(C) **HMB-45 and **(D) **MELAN A were also diffusely positive.

Immunohistochemical staining revealed strong, diffuse positive staining for S-100 protein and positive staining for the melanoma markers HMB45 and Melan-A. Staining for vimentin was also positive. Staining for the epithelial markers cytokeratin (CAM 5.2) and epithelial membrane antigen and the neuroendocrine markers chromogranin A and synaptophysin were negative. A diagnosis of non-pigmented melanoma was established (Figure 2).

After the patient's release from our hospital, an exhaustive examination was conducted by dermatologists, ophthalmologists and oncologists, who found no evidence of lesions suggestive of melanoma in any part of her body. The patient was treated and her records were reviewed by the oncology department. Her treatment consisted of interferon-α-2b (IFNα2b) administered intravenously. Analytical and radiological reviews (abdominal CT scan and chest X-ray) were carried out. Thirty-two abdominal CT scans were obtained throughout this period, making it possible to diagnose and treat relapses in a relatively timely manner.

**Figure 3:**
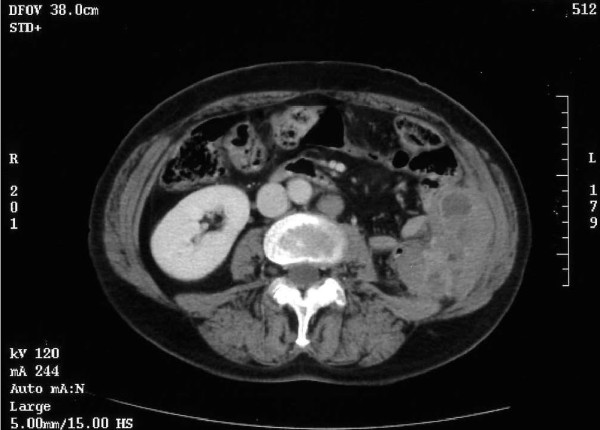
**Image showing a solid mass measuring 7.2 cm × 4 cm with areas of necrosis, situated in the retroperitoneum and invading the wall of the abdomen, in contact with the intestinal loops**. The para-aortic adenopathy measured 1.8 cm.

Two years after the patient's diagnosis and surgery, during a routine review involving an abdominal CT scan, a solid retroperitoneal mass with areas of necrosis measuring 7 cm × 2 cm × 4.5 cm was found in the left renal fossa, invading the abdominal wall and in direct contact with the intestinal loops. Also, a para-aortic adenopathy with a diameter of 1.8 cm was observed (Figure 3).

The patient once again underwent surgery while under general anesthesia. We performed a mid-line suprainfraumbilical incision and located a tumor in the previous surgical area. Its diameter was approximately 10 cm, it seemed to infiltrate the abdominal wall and it was adhered to the splenic angle of the colon, although without infiltration. After the adhesions were removed, we completely removed the tumor, together with a para-aortic adenopathy with a diameter of approximately 3 cm. The post-operative period progressed without any incidents. The pathology of the retroperitoneal lesion and the lymph node showed a tumor entirely similar to the one previously excised.

Four years after the initial diagnosis, during a radiological review (chest X-ray and CT scan), pulmonary nodules were detected that were suggestive of pulmonary metastasis. Chemotherapy with dacarbazine and IFNα2b was begun by the oncology service, and one year later the CT scan revealed that the nodules had disappeared.

**Figure 4:**
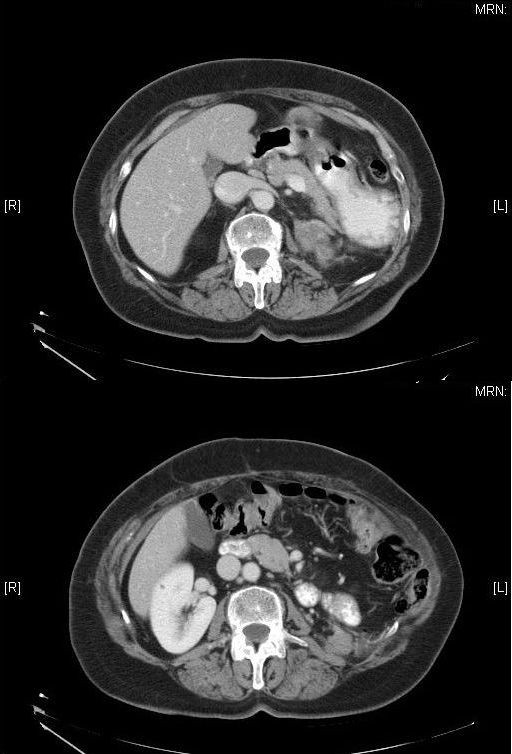
**Image showing a lesion measuring 2.3 cm × 4 cm situated behind and beneath the tail of the pancreas and a lesion measuring 2.5 cm × 2.5 cm situated in the mesenteric fat adjacent to the medial border of the descending colon, compatible with tumor implants**.

In the CT scan obtained eight years after the initial diagnosis, we observed a nodule measuring 4.5 cm × 2.3 cm in the left renal fossa and a nodule measuring 2.5 cm × 2.5 cm in the mesenteric fat of the descending colon (Figure 4). On the basis of these findings, we decided to perform surgery with the patient under general anesthesia to remove the nodules through a left subcostal incision, including the tail of the pancreas and the splenic angle of the colon, re-establishing intestinal continuity by colocolic anastomosis. The tumor remained unchanged in its microscopic appearance. During the subsequent clinical and radiological follow-up, no relapse was observed.

## Discussion

Primary melanoma is a tumor that is most frequently found on the skin and which, on very rare occasions, may originate in the eye (choroid plexus). Melanomas have also been found in the mouth cavity, larynx and bronchi, the esophagus, the rectum, the genitourinary system, the meninges, the ovaries, the uterine cervix, the vagina and the adrenal glands. One of the major obstacles for clinicians is identifying whether the tumor is really a primary tumor or whether it is metastatic [13].

The possibility exists that a tumor lesion of melanocytic cells in the skin or eyes may have gone unnoticed or that a skin lesion removed months or years ago may have been the primary origin of undiagnosed melanoma. Also, some authors have mentioned that primary skin melanoma occasionally reappears spontaneously, and the only clinical symptoms in some of these patients are due to the presence of metastasis in the regional lymphatic ganglia or the internal organs [16-19]. Some authors have even described the total or partial spontaneous regression of melanoma metastasis after bilateral adrenalectomy, which may be partly explained by the fact that human melanoma cells have a high affinity for glucocorticoid receptors. For this reason, adrenalectomy has been shown to have anti-melanoma effects in animals [19].

The adrenal medullary blasts and melanoblasts have a common embryological origin at the neuroectodermal level [20], similarly to the nervous system and the skin. As a result, ectopic melanocytes can exist in this location and cause metaplasia and malignant transformation, leading to the appearance of a melanoma. This would explain why primary melanoma can originate in the adrenal glands. Primary melanoma of the adrenal gland raises the need for a differential diagnosis, especially in cases of metastatic melanoma and pigmented pheochromocytoma. Pigmented adrenal adenoma, adrenal hematoma and adrenocortical carcinoma have to be considered in the differential diagnosis, too.

In primary melanomas, the four stages of melanogenesis are usually observed [21]. With regard to the differential diagnosis of pigmented pheochromocytoma, immunohistochemical studies are necessary, essentially with the neuroendocrine markers chromogranin and synaptophysin, and electron microscopy is also required [11]. Pigmented pheochromocytoma shows immunoreactivity for chromogranin, synaptophysin and enolase. In one case series, it was found that in one-third of the cases of pheochromocytoma, there were HMB45-positive cells, which comes as no surprise because the cells of the adrenal medulla or pheochromocytes are derived from the neural crest in the same way as melanocytes, and HMB45 antibody stains tumors derived from the neural crest. In the pheochromocytoma, there are a number of sustentacular cells on the periphery of the tumor nests, which are stained with S-100. No positivity has been described for Melan-A. By using an electron microscope, neurosecretory granules can be detected in the cytoplasm. In melanoma, the cells are not stained with chromogranin or synaptophysin, and neither is there positivity for catecholamines, enolase and opioid peptides, which are typical pheochromocytoma markers. Melanoma tumor cells are stained with Melan-A and also with HMB45 and S-100. By using an electron microscope, pre-melanosomes or melanosomes can be detected [8-22].

Because at the macroscopic level the melanoma may have a brown or black color, it is necessary to also establish the differential diagnosis with other pigmented adrenal lesions such as pigmented adrenal adenomas [23]. These are tumors of the adrenal cortex, which are benign and small, usually measuring less than 5 cm. Microscopic and immunohistochemical studies lead to the correct diagnosis. These tumors are usually positive for low-molecular-weight keratins, but not for the markers S-100 and HMB45 [23].

At the macroscopic level, adrenal hematomas may also give rise to confusion; however, under the microscope, hemosiderin-laden macrophages may be seen, and the histological characteristics are completely different. Adrenocortical carcinomas are tumors that require the establishment of a differential diagnosis. It is a larger-sized tumor than the adenoma, and its cells do not stain positively for S-100 or HMB45 [23].

## Conclusions

The clinical case we present herein meets the criteria of Ainsworth *et al. *[14] and Carstens *et al. *[4]. The tumor affected a single gland (the left adrenal gland), melanoma was found to be absent in the rest of the organism based on exhaustive dermatological, digestive, ophthalmological and urological examinations, and there was no history of the removal of pigmented lesions. The relapses that were treated surgically were always locoregional, and from a histological and immunohistochemical perspective, they had the characteristics of melanoma. Also, the fact that 11 years have passed since the diagnosis without any evidence of melanoma in any other location leads us to the conclusion that our patient's lesion was a primary melanoma of the adrenal gland.

## Consent

Written informed consent was obtained from the patient for publication of this case report and any accompanying images. A copy of the written consent is available for review by the Editor-in-Chief of this journal.

## Competing interests

The authors declare that they have no competing interests.

## Authors' contributions

LGS participated surgical treatment, design and drafted manuscript. SPF participated in the design, literature review and drafted manuscript. MJLP participated in histopathology immunohistochemical study. FAM participated in histopathology and immunohistochemical study. JMS participated in surgical treatment. JARG participated in surgical treatment. All authors read and approved the final manuscript.
